# Cardiorespiratory fitness is associated with physical literacy in a large sample of Canadian children aged 8 to 12 years

**DOI:** 10.1186/s12889-018-5896-5

**Published:** 2018-10-02

**Authors:** Justin J. Lang, Jean-Philippe Chaput, Patricia E. Longmuir, Joel D. Barnes, Kevin Belanger, Grant R. Tomkinson, Kristal D. Anderson, Brenda Bruner, Jennifer L. Copeland, Melanie J. Gregg, Nathan Hall, Angela M. Kolen, Kirstin N. Lane, Barbi Law, Dany J. MacDonald, Luc J. Martin, Travis J. Saunders, Dwayne Sheehan, Michelle R. Stone, Sarah J. Woodruff, Mark S. Tremblay

**Affiliations:** 1 Healthy Active Living and Obesity (HALO) Research Group, CHEO Research Institute, 401 Smyth Road, Ottawa, ON K1H 8L1 Canada; 2 Department of Kinesiology and Public Health Education, University of North Dakota, Grand Forks, ND 58202 USA; 3 Alliance for Research in Exercise, Nutrition and Activity (ARENA), School of Health Sciences & Sansom Institute for Health Research, University of South Australia, GPO Box 2471, Adelaide, SA Australia; 4 Centre for Sport and Exercise Education, Camosun College, Victoria, BC V9E 2C1 Canada; 5 School of Physical and Health Education, Nipissing University, North Bay, ON P1B 8L7 Canada; 6 Department of Kinesiology and Physical Education, University of Lethbridge, Lethbridge, AB T1K 3M4 Canada; 7 Department of Kinesiology and Applied Health, University of Winnipeg, Winnipeg, MB B2G 0W5 Canada; 8 Department of Human Kinetics, St. Francis Xavier University, Antigonish, NS B2G 0W5 Canada; 9 Department of Applied Human Sciences, University of Prince Edward Island, Charlottetown, PE C1A 4P3 Canada; 10 School of Kinesiology and Health Studies, Queen’s University, Kingston, ON K7L 3N6 Canada; 11 Faculty of Health, Community and Education, Mount Royal University, Calgary, AB T3E 6K6 Canada; 12 School of Health and Human Performance, Dalhousie University, Halifax, NS B3H 4R2 Canada; 13 Department of Kinesiology, University of Windsor, Windsor, ON N9B 3P4 Canada

**Keywords:** Physical fitness, Physical activity, Cognition, Motivation, Confidence, Knowledge, Understanding

## Abstract

**Background:**

The associations between cardiorespiratory fitness (CRF) and physical literacy in children are largely unknown. The aim of this study was to assess the relationships between CRF, measured using the 20-m shuttle run test (20mSRT), and components of physical literacy among Canadian children aged 8–12 years.

**Methods:**

A total of 9393 (49.9% girls) children, with a mean (SD) age of 10.1 (±1.2) years, from a cross-sectional surveillance study were included for this analysis. The SRT was evaluated using a standardized 15 m or 20 m protocol. All 15 m SRTs were converted to 20mSRT values using a standardized formula. The four domains of physical literacy (Physical Competence, Daily Behaviour, Motivation and Confidence, and Knowledge and Understanding) were measured using the Canadian Assessment of Physical Literacy. Tertiles were identified for 20mSRT laps, representing low, medium, and high CRF for each age and gender group. Cohen’s *d* was used to calculate the effect size between the low and high CRF groups.

**Results:**

CRF was strongly and favourably associated with all components of physical literacy among school-aged Canadian children. The effect size between low and high CRF tertile groups was large for the Physical Competence domain (Cohen’s *d* range: 1.11–1.94) across age and gender groups, followed by moderate to large effect sizes for Motivation and Confidence (Cohen’s *d* range: 0.54–1.18), small to moderate effect sizes for Daily Behaviour (Cohen’s *d* range: 0.25–0.81), and marginal to moderate effect sizes for Knowledge and Understanding (Cohen’s *d* range: 0.08–0.70).

**Conclusions:**

This study identified strong favourable associations between CRF and physical literacy and its constituent components in children aged 8–12 years. Future research should investigate the sensitivity and specificity of the 20mSRT in screening those with low physical literacy levels.

**Electronic supplementary material:**

The online version of this article (10.1186/s12889-018-5896-5) contains supplementary material, which is available to authorized users.

## Background

Physical literacy (PL) has emerged as a core construct in the physical education paradigm, designed to support children’s engagement in physical activity throughout the life course [1, 2]. To better understand the state of PL among Canadian children, an 11-site cross-sectional surveillance study called the Royal Bank of Canada–Canadian Assessment of Physical Literacy Learn to Play project (RBC–CAPL) was conducted. The validated CAPL instrument was specifically chosen to measure each of the four domains that describe PL (i.e., Physical Competence, Daily Behaviour, Motivation and Confidence, and Knowledge and Understanding) [3, 4]. The CAPL can be used as a surveillance tool to understand children’s overall PL levels and to identify the PL domains that may require targeted interventions. However, emerging issues with utilizing the CAPL in school-based settings include the amount of time required to assess large groups of children (approximately 90 min with five appraisers to test 25 children), and the fact that teachers may not have the expertise or available resources to conduct a full CAPL assessment.

The 20-m shuttle run test (20mSRT) [5, 6], a reliable and valid assessment of cardiorespiratory fitness (CRF) among children [7, 8], is a measure that informs the Physical Competence domain of the CAPL. The 20mSRT lends itself well to population-based surveillance because it is easy to administer, requires minimal equipment, and can be used to assess large groups of children simultaneously [9]. Furthermore, several studies have identified favourable associations between 20mSRT performance and aspects of PL among children and youth, including physical fitness [10], daily physical activity [11], cognitive ability [12, 13], and psychosocial health [14, 15].

To our knowledge, until now no study has assessed the associations between CRF and overall/domain-specific PL among children. Specifically, assessing the relationship between CRF and the four domains of PL could provide further insight into the importance of CRF, as well as potential strategies for PL screening [2]. The RBC-CAPL provides an opportunity to assess these associations in a large sample of Canadian children, and to quantify the extent to which CRF relates to various components of PL.

The main objective of this study was to evaluate the associations between 20mSRT performance (i.e., CRF) and each component of PL assessed using the CAPL in a large sample of Canadian school-aged children (8–12 years).

## Methods

### Study design

The RBC-CAPL was a cross-sectional surveillance study that took place between 2014 and 2016, and was designed to evaluate the PL levels of Canadian children using a standardized data collection protocol. The study design included 11 data collection sites from seven Canadian provinces: Victoria, British Columbia; Lethbridge, Alberta; Calgary, Alberta; Winnipeg, Manitoba; North Bay, Ontario; Windsor, Ontario; Ottawa, Ontario; Trois-Rivières, Québec; Halifax, Nova Scotia; Antigonish, Nova Scotia; and Charlottetown, Prince Edward Island. The aim was to recruit up to 1300 participants per site over a 3-year data collection period. Each site was also tasked with recruiting an appropriate geographic mix of participants (minimum of 50% of the sample from urban locations and minimum of 20% from rural locations), while attempting to recruit a balance of participants across socio-economic strata. Ethics approval for this project was originally obtained from the Children’s Hospital of Eastern Ontario Research Ethics Board (Ottawa, Ontario; coordinating centre). Each site subsequently obtained approval from their respective research ethics board. Consent and ethics approval were also obtained from all participating school boards, summer camps, community centres, and/or sport leagues. Written informed consent was obtained from parents or legal guardians, and participating children provided verbal assent.

### Participants

Participant recruitment locations were selected across all sites using purposive, non-randomized sampling. Elementary schools across all sites were the primary participant recruitment locations, while summer camps, community centres, and sport leagues were the secondary participant recruitment locations. Participants were considered eligible for this study if they were between the ages of 8.0 and 12.9 years (grades 4–6) and if they were able to participate in maximal effort exercise (i.e., high-intensity exercise). All eligible participants were invited to participate in this study, and potential participants were able to opt out at any time and for any reason, without consequence.

Of the 10,030 participants who took part in RBC-CAPL, a total of 9393 remained in the present analysis after participants without a 20mSRT score (*n* = 637) were excluded. Body mass index across age and gender groups of those excluded from the analysis did not differ significantly (range of *p* values: 0.13–0.84) from those included in the present analysis. Physical activity levels were significantly lower (*p* < 0.006) for 8-year-olds who were excluded from the analysis (*n* = 30) due to missing data, but there were no differences (range of *p* values: 0.07–0.49) for children aged 9–12 years. Among girls, the included sample had a high percentage of healthy CRF levels (mean percentage: 98.4[±0.2]), but these percentages were much lower among boys (mean percentage: 64.3[±0.7]) when using the interim international CRF standards proposed by Ruiz et al. (see Additional file 1) [16, 17].

### Data collection procedures

All data collection staff had a background in fitness or physical activity assessment, and each site’s coordinators were subsequently trained by research staff from the coordinating centre (Ottawa, Ontario). Data collection procedures followed the published CAPL protocol [3, 4], which provides standardized procedures to collect data across the four PL domain.

### Independent variable

CRF was assessed using the 15 m or 20mSRT protocols [5, 6]. The 15 m protocol was used only if there was not enough space to carry out the full 20 m protocol. All children were asked to run back and forth between two parallel lines, 15 m or 20 m apart, following the pace of an audio signal that began at a speed of 8.5 km/h and increased by 0.5 km/h at every 1-min interval. Participants were encouraged at all times to run a maximal effort test. The total number of laps (shuttles) completed was recorded for each participant, and all data from the 15 m protocol were converted to the 20 m protocol using a conversion chart, which was shown to have good classification agreement [18]. Researchers used indoor gymnasiums as the primary testing location, with outdoor locations used as a back-up location when necessary. Following the Tomkinson recommendations [19], 20mSRT performance for this study was reported as the running speed at the last completed stage and number of laps completed.

### Dependent variables

#### Physical literacy

PL was assessed using the CAPL instrument, which provides methods to assess the four domains of PL, as described below. The total CAPL score is an aggregate that combines all domains (Physical Competence [maximum of 32 points], Daily Behaviour [maximum of 32 points], Motivation and Confidence [maximum of 18 points], and Knowledge and Understanding [maximum of 18 points]), and ranges from 0 points (poor PL) to 100 points (excellent PL) [3]. The total CAPL score and each of the four domain scores were used to summarize the associations between CRF and PL.

#### Physical competence

The Physical Competence domain was modified from the original CAPL methods to provide an aggregate score that excluded the 20mSRT. Thus, the Physical Competence domain included three health-related fitness assessments, three anthropometric assessments, and one gross motor movement skill assessment.

*Grip strength* was assessed using a handgrip dynamometer following established procedures [20]. The better score from two trials from each of the left and right hands, measured to the nearest 0.5 kg, were combined. The prone plank test was used to assess *torso muscular endurance* [21]. Participants were asked to hold a static prone position on their elbows and toes with a straight body position from the ankles to the head for as long as possible, with the time to exhaustion (nearest 0.1 s) recorded as the final score. *Flexibility* was assessed using the sit-and-reach protocol with a flexometer [20]. Participants were asked to remove their shoes and then sit with their legs stretched out in front of them and their knees flat on the floor. They were asked to extend their arms with their hands stacked while bending forward at the hips and keeping legs straight. The furthest distance attained while reaching forward toward their toes was recorded to the nearest 0.5 cm.

*Waist circumference* was measured to the nearest 0.5 cm at the top of the iliac crest, using standardized procedures [20]. *Standing height* was assessed to the nearest 0.1 cm using a stadiometer, and *body weight* was recorded to the nearest 0.1 kg using a digital weighing scale. *Body mass index* was calculated from the measured height and weight values (kg/m^2^).

*Gross motor movement skills* were assessed using the Canadian Agility and Movement Skill Assessment (CAMSA) protocol [22]. The CAMSA is a standardized agility course that provides a method to rapidly assess fundamental and complex movement skills (jumping, sliding, catching, throwing, skipping, hopping, and kicking) in a way that incorporates various ‘real-world’ movement capacities (coordination, balance, precision, acceleration, and deceleration). The overall CAMSA score combines movement quality scores with the obstacle course completion time to provide an overall score between 1.5 (low performer) and 42 (high performer) [22].

#### Daily behaviour

The Daily Behaviour domain assessed participants’ engagement in physical activity and sedentary behaviours as three separate components: objective physical activity, self-reported physical activity, and self-reported screen time. *Objective physical activity* was assessed as the average number of steps taken each day using an SC-StepRx pedometer (StepsCount, Deep River, ON, Canada) [23]. Participants were asked to record their daily step counts on a pedometer tracking log before bedtime over the course of seven days. They were also asked to record the time (hour/minute) that they put on and took off the pedometer, and if the pedometer was removed during the day, the amount of time missed and the reason for the removal (e.g., forgot to wear, water-related activity). Pedometer data were considered valid only if three criteria were met: the total number of steps per day was between 1000 and 30,000; at least 10 h of wear time were accumulated per day; and at least three valid days were recorded [23, 24].

Children were asked to self-report the average number of days per week that they performed at least 60 min of moderate- to vigorous-intensity *physical activity*. To measure *sedentary behaviour,* children were asked to self-report the time they spent using screens (i.e., watching television, and playing video games, computer games, or other screen-based devices) on a typical school day and weekend day [25].

#### Motivation and confidence

The Motivation and Confidence domain was derived from five components. A published scale was used to derive participants’ perceptions of physical activity benefits and barriers [26]. Children were asked how their activity levels compared with their peers, and how their skill level compared with their peers. Lastly, sub-scales of the Children’s Self-Perception and Adequacy in and Predilection for Physical Activity (CSAPPA) scale were used to assess participants’ self-reported adequacy, as well as their self-reported predilection, toward physical activity participation [26, 27].

#### Knowledge and understanding

Participants’ Knowledge and Understanding was assessed using a standardized questionnaire (CAPL questionnaire) developed to reflect the Canadian curricula for physical and health education for grades 4, 5, and 6 [3]. The questions were broadly related to the understanding of the sedentary behaviour and physical activity guidelines [28, 29], health-related fitness components, physical activity safety equipment, and methods for improving movement skills. Participants completed the questionnaire using paper and pencil or an online website format.

### Maturity offset

To control for the impact of maturation, maturity offset was calculated from age (years) and standing height (cm) using the following equations [30]:

$$ For\ boys: Maturity\ offset=-7.999994+\left({0.0036124}^{\ast}\left({age}^{\ast } height\right)\right);{R}^2=0.896; SEE=0.542 $$

$$ For\ girls: Maturity\ offset=-7.709133+\left({0.0042232}^{\ast}\left({age}^{\ast } height\right)\right);{R}^2=0.898; SEE=0.528 $$

### Data analysis

All analyses were conducted using SAS 9.4 (SAS Institute Inc., Cary, North Carolina, USA). Descriptive characteristics for all variables were calculated as means and standard deviations. To determine the analytical plan to assess the associations between CRF and each dependent variable, age and gender interaction terms were tested. First, significant gender interactions were identified between CRF and most components of PL (i.e., 16/20 significant interactions). Next, significant age interactions by gender were identified for more than half of the components of PL (i.e., 24/36 significant interactions). As a result of these significant interactions, a stratified analysis by age and gender was conducted. The independent variable, 20mSRT laps, was divided into tertiles across each age (i.e., age at the last birthday) by gender group, representing low, medium, and high CRF. Thus, mean scores for all components of PL were calculated for each age by gender CRF tertile group. Levene’s test was used to assess the equality of variance. Significant differences across groups were assessed using analysis of covariance (ANCOVA), controlling for predicted maturity offset [30]. Post-hoc analyses (Dunn’s test with Bonferroni correction) were used to identify significant differences between groups, using low CRF as the reference group. Effect sizes between low and high CRF groups were calculated using Cohen’s *d*, and effect sizes were interpreted as small (*d* between 0.2 and 0.5), moderate (*d* between 0.5 and 0.8), and large (*d* > 0.8), with effect sizes below 0.2 considered trivial [31].

## Results

### Participants

A total of 9393 (49.9% girls) children aged 8–12 years, from 11 Canadian sites, were included in this study. Nearly half (42.6%) of the sample was from Western Canada (i.e., Victoria, Lethbridge, Calgary, and Winnipeg). Table 1 provides the descriptive characteristics for all included participants.

**Table 1:** Descriptive characteristics of participants

	Boys (*n* = 4710)	Girls (*n* = 4683)	Total (*n* = 9393)
Age (years)	10.1 (1.2)	10.1 (1.2)	10.1 (1.2)
Maturity offset (predicted from age (years) and standing height (cm)) [30]	−2.7 (0.9)	−1.5 (1.1)	− 2.1 (1.2)
Site (n, %)			
Victoria, British Columbia	258 (5.5)	220 (4.7)	478 (5.1)
Lethbridge, Alberta	532 (11.3)	531 (11.3)	1063 (11.3)
Calgary, Alberta	620 (13.2)	619 (13.2)	1239 (13.2)
Winnipeg, Manitoba	607 (12.9)	610 (13.0)	1217 (13.0)
North Bay, Ontario	529 (11.2)	578 (12.3)	1107 (11.8)
Windsor, Ontario	637 (13.5)	569 (12.2)	1206 (12.8)
Ottawa, Ontario	350 (7.4)	359 (7.7)	709 (7.5)
Trois-Rivières, Québec	56 (1.2)	34 (0.7)	90 (1.0)
Halifax, Nova Scotia	390 (8.3)	407 (8.7)	797 (8.5)
Antigonish, Nova Scotia	482 (10.2)	506 (10.8)	988 (10.5)
Charlottetown, P.E.I.	249 (5.3)	250 (5.3)	499 (5.3)
Independent variable			
20mSRT (# laps)	25.8 (15.8)	20.9 (11.6)	23.4 (14.1)
Running speed at the last completed stage (km/h)^a^			
8-year-olds	8.9 (2.1)	8.8 (1.6)	8.8 (1.8)
9-year-olds	9.1 (1.6)	8.9 (1.6)	9.0 (1.6)
10-year-olds	9.2 (1.5)	9.0 (1.3)	9.1 (1.4)
11-year-olds	9.4 (1.5)	9.2 (1.2)	9.3 (1.3)
12-year-olds	9.5 (1.6)	9.3 (1.2)	9.4 (1.4)
Dependent variables			
Physical Literacy			
Total CAPL score (0–100)	63.9 (12.4)	63.7 (10.9)	63.8 (11.7)
Physical Competence domain			
Total domain score (0–32)	20.8 (4.3)	20.8 (4.2)	20.8 (4.2)
Handgrip score (kg)	34.5 (9.6)	32.6 (9.2)	33.6 (9.5)
Prone plank score (sec)	62.0 (44.2)	61.3 (42.7)	61.5 (43.5)
Sit-and-reach score (cm)	25.4 (7.6)	30.9 (8.3)	28.1 (8.4)
Waist circumference (cm)	67.4 (11.0)	67.2 (10.6)	67.3 (10.8)
Body mass index (kg/m^2^)	18.9 (3.9)	19.0 (3.8)	19.0 (3.8)
CAMSA score	31.5 (5.9)	30.4 (5.7)	31.0 (5.8)
Daily Behaviour domain			
Total domain score (0–32)	18.6 (7.9)	18.4 (7.4)	18.5 (7.6)
Average daily step counts	12,405 (4081)	10,793 (3508)	11,530 (3865)
Self-reported screen time (h)	2.7 (2.1)	2.2 (1.8)	2.5 (1.9)
Self-reported physical activity (days/week meeting the guidelines)	5.0 (2.0)	4.9 (1.9)	5.0 (1.9)
Motivation and Confidence domain			
Total domain score (0–18)	12.7 (2.8)	12.2 (2.6)	12.5 (2.7)
Benefits and barriers	1.6 (1.2)	1.5 (1.1)	1.6 (1.2)
Activity levels compared to peers	0.7 (0.2)	0.7 (0.2)	0.7 (0.2)
Skill level compared to peers	0.7 (0.2)	0.6 (0.2)	0.7 (0.2)
CSAPPA adequacy score	4.8 (0.9)	4.6 (0.9)	4.7 (0.9)
CSAPPA predilection score	4.8 (1.0)	4.8 (1.0)	4.8 (1.0)
Knowledge and Understanding domain			
Total domain score (0–18)	11.8 (2.8)	12.2 (2.6)	12.0 (2.7)
CAPL questionnaire score	11.8 (2.8)	12.2 (2.6)	12.0 (2.7)

Note: Variables are presented as means (±SD) unless otherwise stated

*20mSRT* 20-m shuttle run test, *CAMSA* Canadian Agility and Movement Skill Assessment, *CAPL* Canadian Assessment of Physical Literacy, *CSAPPA* Children’s Self-Perception and Adequacy in and Predilection for Physical Activity

^a^20mSRT results are reported as running speed (km/h) at the last completed stage and number of laps completed for each age group as recommended by Tomkinson et al. [19]

### Associations across components of physical literacy

Tables 2, 3, 4, 5 and 6 display the associations between CRF and all components of PL by age, with each table presenting the data for boys and girls of the same age. There were significant main effects across CRF tertile groups for most components of PL that were consistent and in the expected direction across all age and gender groups. The effect sizes (Cohen’s *d*) between low and high CRF groups ranged from small to large, with very few components of PL considered trivial. Generally, larger effect sizes were observed for boys than girls, and the effect sizes generally increased with age. The Physical Competence domain score consistently displayed the largest effect size (Cohen’s *d* range: 1.11–1.94) across age and gender groups, followed by the total CAPL score (Cohen’s *d* range: 0.92–1.60), the CAMSA (Cohen’s *d* range: 0.97–1.52) and the plank (Cohen’s *d* range: 0.86–1.36). The smallest effect size across age and gender groups was generally the Knowledge and Understanding domain (Cohen’s *d* range: 0.08–0.70), followed by the sit and reach score for boys (Cohen’s *d* range: 0.29–0.40), and the handgrip score for girls (Cohen’s *d* range: 0.24–0.41).

**Table 2:** Tertiles of cardiorespiratory fitness across components of physical literacy for 8-year-old boys and girls

20mSRT (# laps)	Boys (*n* = 522)				Girls (*n* = 524)			
	low CRF	medium CRF	high CRF	Cohen’s d	low CRF	medium CRF	high CRF	Cohen’s d
	9.4 (2.7)	19.5 (3.6)	39.5 (11.2)		9.5 (2.1)	15.9 (1.9)	31.1 (10.6)	
Physical Literacy								
Total CAPL score	55.1 (10.8)	62.1 (10.3)^a^	67.7 (9.8)^b,c^*	*1.22*	58.7 (9.9)	62.7 (9.9)^a^	67.1 (8.3)^b,c^*	*0.92*
Physical Competence domain								
Total domain score	17.5 (3.4)	20.0 (3.2)^a^	22.4 (3.0)^b,c^*	*1.53*	17.4 (3.8)	19.8 (3.2)^a^	21.3 (3.2)^b,c^*	*1.11*
Handgrip score (kg)	25.5 (6.8)	26.8 (5.6)	29.3 (5.6)^b,c^*	*0.61*	24.5 (5.9)	24.9 (5.4)	26.8 (5.3)^b,c^*	*0.41*
Prone plank score (sec)	37.6 (27.2)	51.9 (34.5)^a^	72.0 (41.5)^b,c^*	*0.98*	39.3 (21.6)	54.9 (37.3)^a^	74.0 (50.6)^b,c^*	*0.89*
Sit-and-reach score (cm)	26.1 (6.7)	27.1 (7.0)	28.1 (6.8)^c^*	*0.30*	29.8 (7.0)	32.0 (6.5)^a^	32.1 (7.5)^c^*	*0.32*
Waist circumference (cm)	63.2 (9.0)	60.8 (6.9)^a^	59.0 (4.4)^b,c^*	*0.59*	64.4 (9.8)	60.8 (7.7)^a^	59.2 (6.6)^c^*	*0.62*
BMI (kg/m^2^)	18.1 (3.4)	16.9 (2.3)^a^	16.6 (1.9)^c^*	*0.55*	18.3 (3.5)	16.9 (2.5)^a^	16.7 (2.5)^c^*	*0.53*
CAMSA score	23.7 (6.0)	28.5 (5.3)^a^	31.2 (5.1)^b,c^*	*1.35*	23.5 (5.7)	26.3 (5.7)^a^	28.9 (5.4)^b,c^*	*0.97*
Daily Behaviour domain								
Total domain score	16.3 (7.9)	19.3 (7.3)^a^	21.6 (6.9)^b,c^*	*0.71*	18.8 (7.4)	20.2 (7.1)	21.9 (6.4)^c^*	*0.45*
Average daily step counts	11,369 (3786)	13,589 (3907)^a^	14,218 (3471)^c^*	*0.78*	11,270 (3479)	11,773 (3242)	11,758 (3283)	*0.14*
Self-reported screen time (h/day)	3.2 (2.4)	2.4 (1.8)^a^	2.1 (2.0)^c^*	*0.50*	2.2 (2.0)	1.9 (1.8)	1.6 (1.5)^c^	*0.34*
Average days/week meeting the guidelines	4.4 (2.3)	4.7 (2.4)	5.4 (1.9)^b,c^*	*0.47*	4.5 (2.3)	4.8 (2.1)	5.2 (1.8)^c^	*0.34*
Motivation and Confidence domain								
Total domain score	11.5 (2.8)	12.3 (2.5)^a^	13.2 (2.3)^b,c^*	*0.66*	12.0 (2.3)	11.9 (2.5)	13.2 (2.1)^b,c*^	*0.54*
Benefits and barriers	1.3 (1.4)	1.5 (1.3)	1.8 (1.2)^c^*	*0.38*	1.4 (1.2)	1.3 (1.2)	1.6 (1.1)^c^	*0.17*
Activity levels compared to peers	0.7 (0.3)	0.7 (0.2)	0.8 (0.2)^c^*	*0.39*	0.7 (0.2)	0.7 (0.2)	0.8 (0.2)^b^*	*0.50*
Skill level compared to peers	0.7 (0.3)	0.7 (0.3)	0.8 (0.2)^b,c^*	*0.39*	0.7 (0.2)	0.7 (0.2)	0.7 (0.2)	*0.00*
CSAPPA adequacy score	4.5 (0.9)	4.6 (0.8)	4.9 (0.8)^b,c^*	*0.47*	4.5 (0.9)	4.5 (0.8)	4.9 (0.8)^b,c^*	*0.47*
CSAPPA predilection score	4.3 (1.0)	4.8 (0.9)^a^	5.0 (0.9)^c^*	*0.74*	4.6 (0.9)	4.8 (0.9)	5.2 (0.7)^b,c^*	*0.74*
Knowledge and Understanding domain								
Total domain score	9.8 (2.7)	10.5 (3.0)	10.4 (2.6)	*0.23*	10.5 (2.5)	10.7 (2.6)	10.7 (2.6)	*0.08*
CAPL questionnaire score	9.8 (2.7)	10.5 (3.0)	10.4 (2.6)	*0.23*	10.5 (2.5)	10.7 (2.6)	10.7 (2.6)	*0.08*

Note: All variables are presented as means (±SD). Statistical significance for the main effect was assessed using ANCOVA controlling for maturity offset. Bonferroni (Dunn) t-test was used to assess differences between groups. Cohen’s *d* was used to calculate the effect size between the low and high CRF groups

*20mSRT* 20-m shuttle run test, *ANCOVA* analysis of covariance, *BMI* body mass index, *CAMSA* Canadian Agility and Movement Skill Assessment, *CAPL* Canadian Assessment of Physical Literacy, *CRF* cardiorespiratory fitness, *CSAPPA* Children’s Self-Perception and Adequacy in and Predilection for Physical Activity

^a^*p* < 0.05 for medium CRF vs low CRF

^b^*p* < 0.05 for high CRF vs medium CRF

^c^*p* < 0.05 for high CRF vs low CRF

* *p* < 0.001 for main effect

**Table 3:** Tertiles of cardiorespiratory fitness across components of physical literacy for 9-year-old boys and girls

20mSRT (# laps)	Boys (*n* = 946)				Girls (*n* = 908)			
	low CRF	medium CRF	high CRF	Cohen’s d	low CRF	medium CRF	high CRF	Cohen’s d
	10.7 (3.0)	21.0 (3.5)	40.9 (10.7)		9.6 (2.3)	17.2 (2.5)	32.3 (8.5)	
Physical Literacy								
Total CAPL score	57.3 (11.6)	63.9 (10.1)^a^	70.6 (9.7)^b,c^*	*1.24*	59.4 (9.9)	63.4 (10.1)^a^	68.4 (8.4)^b,c^*	*0.98*
Physical Competence domain								
Total domain score	17.5 (3.7)	20.4 (3.4)^a^	22.9 (3.4)^b,c^*	*1.52*	17.6 (3.8)	20.3 (3.5)^a^	22.7 (3.5)^b,c^*	*1.40*
Handgrip score (kg)	29.9 (7.5)	30.3 (7.3)	32.1 (6.9)^b,c^*	*0.31*	27.7 (6.8)	28.5 (6.6)	29.3 (6.3)^c^*	*0.24*
Prone plank score (sec)	41.8 (31.0)	55.8 (36.8)^a^	83.1 (53.7)^b,c^*	*0.94*	44.4 (30.0)	60.1 (41.8)^a^	80.7 (51.3)^b,c^*	*0.86*
Sit-and-reach score (cm)	25.0 (7.9)	26.1 (7.3)	28.0 (7.0)^b,c^*	*0.40*	29.5 (7.7)	31.4 (7.9)^a^	32.1 (7.7)^c^*	*0.34*
Waist circumference (cm)	68.7 (12.0)	63.3 (7.5)^a^	60.6 (5.4)^b,c^*	*0.87*	68.8 (10.7)	64.3 (9.4)^a^	60.4 (7.3)^b,c^*	*0.92*
BMI (kg/m^2^)	20.1 (4.3)	17.7 (2.6)^a^	16.9 (2.0)^b,c^*	*0.95*	19.9 (4.1)	18.2 (3.1)^a^	17.1 (2.6)^b,c^*	*0.82*
CAMSA score	26.8 (5.5)	29.9 (4.7)^a^	33.5 (4.2)^b,c^*	*1.37*	25.9 (5.7)	29.4 (5.0)^a^	32.0 (4.5)^b,c^*	*1.19*
Daily Behaviour domain								
Total domain score	17.3 (8.0)	19.8 (7.5)^a^	22.6 (6.7)^b,c^*	*0.72*	18.9 (7.2)	19.2 (7.3)	20.9 (6.3)^b,c^*	*0.25*
Average daily step counts	11,745 (3751)	12,697 (3805)	14,632 (3982)^b,c^*	*0.75*	10,476 (3486)	11,135 (3386)	11,993 (3380)^b,c^*	*0.44*
Self-reported screen time (h/day)	3.1 (2.6)	2.5 (1.8)^a^	2.0 (1.7)^b,c^*	*0.50*	2.1 (1.9)	2.0 (1.7)	1.7 (1.5)^c^	*0.23*
Average days/week meeting the guidelines	4.5 (2.2)	5.0 (2.1)^a^	5.5 (1.8)^b,c^*	*0.50*	4.7 (2.1)	4.9 (2.0)	5.3 (1.6)^b,c^*	*0.32*
Motivation and Confidence domain								
Total domain score	11.9 (2.8)	12.6 (2.4)^a^	13.8 (2.1)^b,c^*	*0.77*	11.7 (2.6)	12.5 (2.2)^a^	13.2 (2.0)^b,c^*	*0.65*
Benefits and barriers	1.5 (1.3)	1.6 (1.2)	1.8 (1.2)^c^*	*0.24*	1.5 (1.2)	1.6 (1.0)	1.7 (1.0)	*0.18*
Activity levels compared to peers	0.7 (0.2)	0.7 (0.2)^a^	0.8 (0.2)^b,c^*	*0.50*	0.7 (0.2)	0.7 (0.2)^a^	0.8 (0.2)^b,c^*	*0.50*
Skill level compared to peers	0.7 (0.3)	0.7 (0.2)	0.8 (0.2)^b,c^*	*0.39*	0.6 (0.2)	0.7 (0.2)^a^	0.7 (0.2)^c^*	*0.50*
CSAPPA adequacy score	4.5 (0.9)	4.8 (0.8)^a^	5.1 (0.7)^b,c^*	*0.74*	4.3 (0.9)	4.6 (0.8)^a^	4.9 (0.8)^b,c^*	*0.71*
CSAPPA predilection score	4.6 (1.0)	4.8 (0.9)^a^	5.2 (0.8)^b,c^*	*0.66*	4.6 (1.0)	4.8 (0.9)	5.2 (0.8)^b,c^*	*0.66*
Knowledge and Understanding domain								
Total domain score	10.6 (2.8)	11.0 (2.7)	11.3 (2.6)^c^*	*0.26*	11.1 (2.5)	11.4 (2.5)	11.6 (2.3)^c^	*0.21*
CAPL questionnaire score	10.6 (2.8)	11.0 (2.7)	11.3 (2.6)^c^*	*0.26*	11.1 (2.5)	11.4 (2.5)	11.6 (2.3)^c^	*0.21*

Note: All variables are presented as means (±SD). Statistical significance for the main effect was assessed using ANCOVA controlling for maturity offset. Bonferroni (Dunn) t-test was used to assess differences between groups. Cohen’s *d* was used to calculate the effect size between the low and high CRF groups

*20mSRT* 20-m shuttle run test, *ANCOVA* analysis of covariance, *BMI* body mass index, *CAMSA* Canadian Agility and Movement Skill Assessment, *CAPL* Canadian Assessment of Physical Literacy, *CRF* cardiorespiratory fitness, *CSAPPA* Children’s Self-Perception and Adequacy in and Predilection for Physical Activity, *SD* standard deviation

^a^*p* < 0.05 for medium CRF vs low CRF

^b^*p* < 0.05 for high CRF vs medium CRF

^c^*p* < 0.05 for high CRF vs low CRF

*p < 0.001 for main effect

**Table 4:** Tertiles of cardiorespiratory fitness across components of physical literacy for 10-year-old boys and girls

20mSRT (# laps)	Boys (*n* = 1201)				Girls (*n* = 1175)			
	low CRF	medium CRF	high CRF	Cohen’s d	low CRF	medium CRF	high CRF	Cohen’s d
	11.0 (3.0)	21.4 (3.9)	42.6 (10.0)		10.0 (2.1)	16.7 (2.1)	32.0 (9.9)	
Physical Literacy								
Total CAPL score	56.4 (11.4)	63.6 (10.5)^a^	71.9 (9.2)^b,c^*	*1.50*	57.3 (10.2)	63.4 (9.7)^a^	69.6 (9.9)^b,c^*	*1.22*
Physical Competence domain								
Total domain score	17.8 (3.8)	20.5 (3.5)^a^	23.7 (3.4)^b,c^*	*1.64*	17.9 (3.8)	20.4 (3.4)^a^	23.1 (3.3)^b,c^*	*1.46*
Handgrip score (kg)	31.4 (7.9)	32.8 (8.2)^a^	35.7 (7.6)^b,c^*	*0.56*	30.1 (7.7)	31.2 (7.7)^a^	32.8 (7.3)^b,c^*	*0.36*
Prone plank score (sec)	40.7 (31.7)	56.8 (35.3)^a^	85.7 (51.5)^b,c^*	*1.05*	40.5 (28.8)	55.7 (32.6)^a^	80.1 (46.2)^b,c^*	*1.03*
Sit-and-reach score (cm)	24.4 (7.5)	25.7 (7.4)^a^	26.4 (7.4)^c^*	*0.27*	28.6 (7.7)	30.1 (8.0)^a^	32.4 (8.5)^b,c^*	*0.47*
Waist circumference (cm)	72.4 (12.9)	66.6 (9.1)^a^	62.8 (6.5)^b,c^*	*0.94*	72.2 (12.3)	66.5 (8.8)^a^	63.5 (7.7)^b,c^*	*0.85*
BMI (kg/m^2^)	20.6 (4.7)	18.7 (3.2)^a^	17.4 (2.2)^b,c^*	*0.87*	20.7 (4.5)	18.8 (3.2)^a^	17.6 (2.8)^b,c^*	*0.83*
CAMSA score	27.8 (5.8)	31.5 (4.5)^a^	34.7 (4.0)^b,c^*	*1.39*	27.4 (5.3)	29.7 (4.8)^a^	32.9 (4.6)^b,c^*	*1.11*
Daily Behaviour domain								
Total domain score	15.7 (7.6)	18.3 (7.4)^a^	21.5 (6.7)^b,c^*	*0.81*	16.7 (7.1)	18.4 (6.9)^a^	20.5 (7.1)^b,c^*	*0.54*
Average daily step counts	10,923 (3696)	12,327 (3935)^a^	13,907 (4026)^b,c^*	*0.77*	10,065 (3126)	10,771 (3825)	11,569 (3668)^c^*	*0.44*
Self-reported screen time (h/day)	3.3 (2.3)	2.7 (1.9)^a^	2.2 (1.6)^b,c^*	*0.56*	2.4 (1.9)	1.9 (1.5)^a^	1.9 (1.5)^c^*	*0.29*
Average days/week meeting the guidelines	4.5 (2.1)	5.1 (1.9)^a^	5.8 (1.5)^b,c^*	*0.71*	4.5 (1.9)	5.0 (1.9)^a^	5.3 (1.5)^c^*	*0.48*
Motivation and Confidence domain								
Total domain score	11.8 (2.9)	12.8 (2.6)^a^	14.2 (2.1)^b,c^*	*0.95*	11.2 (2.9)	12.2 (2.5)^a^	13.2 (2.2)^b,c^*	*0.78*
Benefits and barriers	1.4 (1.3)	1.6 (1.1)^a^	2.1 (1.1)^b,c^*	*0.58*	1.3 (1.2)	1.4 (1.1)	1.7 (1.0)^b,c^*	*0.36*
Activity levels compared to peers	0.7 (0.2)	0.7 (0.2)^a^	0.8 (0.2)^b,c^*	*0.50*	0.6 (0.2)	0.7 (0.2)^a^	0.7 (0.2)^b,c^*	*0.50*
Skill level compared to peers	0.6 (0.3)	0.7 (0.2)^a^	0.8 (0.2)^b,c^*	*0.78*	0.6 (0.2)	0.6 (0.2)^a^	0.7 (0.2)^b,c^*	*0.50*
CSAPPA adequacy score	4.5 (1.0)	4.9 (0.9)^a^	5.2 (0.7)^b,c^*	*0.81*	4.2 (1.0)	4.6 (0.9)^a^	4.9 (0.8)^b,c^*	*0.77*
CSAPPA predilection score	4.5 (1.1)	4.9 (0.9)^a^	5.3 (0.7)^b,c^*	*0.87*	4.5 (1.1)	4.8 (1.0)^a^	5.2 (0.8)^b,c^*	*0.83*
Knowledge and Understanding domain								
Total domain score	11.4 (2.8)	12.0 (2.6)^a^	12.5 (2.6)^b,c^*	*0.41*	11.6 (2.7)	12.4 (2.4)^a^	12.8 (2.4)^c^*	*0.47*
CAPL questionnaire score	11.4 (2.8)	12.0 (2.6)^a^	12.5 (2.6)^b,c^*	*0.41*	11.6 (2.7)	12.4 (2.4)^a^	12.8 (2.4)^c^*	*0.47*

Note: All variables are presented as means (±SD). Statistical significance for the main effect was assessed using ANCOVA controlling for maturity offset. Bonferroni (Dunn) t-test was used to assess differences between groups. Cohen’s *d* was used to calculate the effect size between the low and high CRF groups

*20mSRT* 20-m shuttle run test, *ANCOVA* analysis of covariance, *BMI* body mass index, *CAMSA* Canadian Agility and Movement Skill Assessment, *CAPL* Canadian Assessment of Physical Literacy, *CRF* cardiorespiratory fitness, *CSAPPA* Children’s Self-Perception and Adequacy in and Predilection for Physical Activity, *SD* standard deviation

^a^*p* < 0.05 for medium CRF vs low CRF

^b^*p* < 0.05 for high CRF vs medium CRF

^c^*p* < 0.05 for high CRF vs low CRF

*p < 0.001 for main effect

**Table 5:** Tertiles of cardiorespiratory fitness across components of physical literacy for 11-year-old boys and girls

20mSRT (# laps)	Boys (*n* = 1510)				Girls (*n* = 1550)			
	low CRF	medium CRF	high CRF	Cohen’s d	low CRF	medium CRF	high CRF	Cohen’s d
	11.5 (3.3)	24.0 (4.6)	47.6 (12.2)		11.6 (2.8)	19.6 (2.6)	35.6 (9.2)	
Physical Literacy								
Total CAPL score	56.4 (11.4)	65.1 (11.0)^a^	72.2 (10.3)^b,c^*	*1.45*	58.0 (10.3)	63.5 (9.7)^a^	70.3 (9.9)^b,c^*	*1.22*
Physical Competence domain								
Total domain score	18.1 (3.8)	21.6 (3.5)^a^	24.4 (3.3)^b,c^*	*1.77*	19.0 (3.6)	21.6 (3.3)^a^	24.0 (3.4)^b,c^*	*1.43*
Handgrip score (kg)	36.0 (9.8)	37.0 (9.1)	39.7 (8.5)^b,c^*	*0.40*	34.6 (9.2)	35.5 (9.0)	37.2 (8.6)^b,c^*	*0.29*
Prone plank score (sec)	42.2 (31.9)	63.0 (39.1)^a^	93.2 (53.1)^b,c^*	*1.16*	43.3 (27.9)	60.0 (37.5)^a^	85.5 (48.5)^b,c^*	*1.07*
Sit-and-reach score (cm)	23.0 (7.6)	25.2 (7.6)^a^	25.8 (7.3)^b^*	*0.38*	28.6 (8.6)	30.4 (8.7)^a^	33.1 (8.6)^b,c^*	*0.52*
Waist circumference (cm)	76.7 (14.1)	69.1 (9.4)^a^	65.3 (6.7)^b,c^*	*1.31*	74.1 (12.3)	68.5 (9.3)^a^	64.8 (7.1)^b,c^*	*0.93*
BMI (kg/m^2^)	21.8 (5.0)	19.2 (3.3)^a^	17.8 (2.4)^b,c^*	*1.01*	21.1 (4.5)	19.3 (3.3)^a^	18.0 (2.7)^b,c^*	*0.84*
CAMSA score	29.7 (5.4)	33.5 (4.5)^a^	36.1 (3.6)^b,c^*	*1.40*	29.5 (5.1)	32.0 (4.4)^a^	34.7 (4.1)^b,c^*	*1.12*
Daily Behaviour domain								
Total domain score	15.2 (7.8)	18.2 (7.5)^a^	20.5 (7.6)^b,c^*	*0.69*	15.6 (7.7)	17.2 (7.0)^a^	19.6 (7.1)^b,c^*	*0.54*
Average daily step counts	10,984 (3740)	11,825 (3865)^a^	13,209 (4118)^b,c^*	*0.57*	9679 (3282)	10,176 (3266)	11,587 (3777)^b,c^*	*0.54*
Self-reported screen time (h/day)	3.4 (2.3)	2.8 (1.9)^a^	2.4 (1.7)^b,c^*	*0.50*	2.7 (2.0)	2.4 (1.7)^a^	2.0 (1.5)^b,c^*	*0.40*
Average days/week meeting the guidelines	4.4 (2.1)	5.1 (1.8)^a^	5.7 (1.6)^b,c^*	*0.70*	4.4 (1.9)	4.9 (1.7)^a^	5.3 (1.6)^b,c^*	*0.51*
Motivation and Confidence domain								
Total domain score	11.3 (3.1)	12.7 (2.8)^a^	14.1 (2.1)^b,c^*	*1.06*	11.1 (2.6)	11.9 (2.4)^a^	13.3 (2.4)^b,c^*	*0.88*
Benefits and barriers	1.3 (1.2)	1.7 (1.2)^a^	2.0 (1.1)^b,c^*	*0.61*	1.3 (1.1)	1.5 (1.0)^a^	1.8 (1.0)^b,c^*	*0.48*
Activity levels compared to peers	0.6 (0.2)	0.7 (0.2)^a^	0.8 (0.2)^b,c^*	*1.00*	0.6 (0.2)	0.7 (0.2)^a^	0.8 (0.2)^b,c^*	*1.00*
Skill level compared to peers	0.6 (0.2)	0.7 (0.2)^a^	0.8 (0.2)^b,c^*	*1.00*	0.6 (0.2)	0.6 (0.2)^a^	0.7 (0.2)^b,c^*	*0.50*
CSAPPA adequacy score	4.4 (1.0)	4.8 (0.9)^a^	5.2 (0.7)^b,c^*	*0.93*	4.2 (0.9)	4.5 (0.8)^a^	4.9 (0.8)^b,c^*	*0.82*
CSAPPA predilection score	4.4 (1.1)	4.8 (1.0)^a^	5.3 (0.8)^b,c^*	*0.94*	4.4 (1.0)	4.6 (0.9)^a^	5.1 (0.8)^b,c^*	*0.77*
Knowledge and Understanding domain								
Total domain score	11.8 (2.8)	12.7 (2.6)^a^	13.1 (2.5)^c^*	*0.49*	12.2 (2.6)	12.8 (2.4)^a^	13.4 (2.4)^b,c^*	*0.48*
CAPL questionnaire score	11.8 (2.8)	12.7 (2.6)^a^	13.1 (2.5)^c^*	*0.49*	12.2 (2.6)	12.8 (2.4)^a^	13.4 (2.4)^b,c^*	*0.48*

Note: All variables are presented as means (±SD). Statistical significance for the main effect was assessed using ANCOVA controlling for maturity offset. Bonferroni (Dunn) t-test was used to assess differences between groups. Cohen’s *d* was used to calculate the effect size between the low and high CRF groups

*20mSRT* 20-m shuttle run test, *ANCOVA* analysis of covariance, *BMI* body mass index, *CAMSA* Canadian Agility and Movement Skill Assessment, *CAPL* Canadian Assessment of Physical Literacy, *CRF* cardiorespiratory fitness, *CSAPPA* Children’s Self-Perception and Adequacy in and Predilection for Physical Activity, *SD* standard deviation

^a^*p* < 0.05 for medium CRF vs low CRF

^b^*p* < 0.05 for high CRF vs medium CRF

^c^*p* < 0.05 for high CRF vs low CRF

*p < 0.001 for main effect

**Table 6:** Tertiles of cardiorespiratory fitness across components of physical literacy for 12-year-old boys and girls

20mSRT (# laps)	Boys (*n* = 531)				Girls (*n* = 526)			
	low CRF	medium CRF	high CRF	Cohen’s d	low CRF	medium CRF	high CRF	Cohen’s d
	12.5 (3.9)	26.6 (4.9)	51.9 (13.1)		12.5 (3.0)	21.0 (2.9)	39.0 (10.9)	
Physical Literacy								
Total CAPL score	55.5 (12.0)	66.7 (11.3)^a^	73.3 (10.2)^b,c^*	*1.60*	58.5 (10.7)	64.7 (10.0)^a^	71.7 (10.3)^b,c^*	*1.26*
Physical Competence domain								
Total domain score	18.4 (4.0)	22.2 (3.5)^a^	25.1 (2.8)^b,c^*	*1.94*	19.5 (3.9)	21.9 (3.8)^a^	24.8 (3.4)^b,c^*	*1.45*
Prone plank score (sec)	42.3 (21.4)	65.9 (34.1)^a^	87.5 (41.9)^b,c^*	*1.36*	44.3 (23.6)	57.1 (35.5)^a^	93.9 (58.7)^b,c^*	*1.11*
Sit-and-reach score (cm)	22.3 (8.1)	24.3 (7.7)	25.2 (7.6)^c^*	*0.37*	31.3 (9.0)	31.1 (8.6)	33.4 (8.8)^b^	*0.24*
Waist circumference (cm)	76.5 (14.2)	70.8 (9.9)^a^	66.0 (6.4)^b,c^*	*0.95*	74.9 (11.5)	71.2 (10.8)^a^	67.9 (7.6)^b,c^*	*0.72*
Handgrip score (kg)	39.5 (11.6)	42.9 (9.7)^a^	46.0 (11.1)^b,c^*	*0.57*	38.6 (11.4)	40.0 (8.9)	42.5 (8.7)^b,c^*	*0.39*
BMI (kg/m^2^)	21.8 (5.1)	19.9 (3.9)^a^	18.1 (2.4)^b,c^*	*0.93*	21.6 (4.8)	20.0 (3.8)^a^	18.6 (2.5)^b,c^*	*0.78*
CAMSA score	29.8 (5.8)	34.3 (4.3)^a^	37.0 (3.4)^b,c^*	*1.52*	30.4 (5.3)	33.3 (4.1)^a^	35.6 (4.4)^b,c^*	*1.07*
Daily Behaviour domain								
Total domain score	14.4 (7.8)	18.3 (8.0)^a^	20.3 (8.0)^b,c^*	*0.75*	15.1 (7.6)	17.1 (7.6)	19.6 (7.4)^b,c^*	*0.60*
Average daily step counts	10,080 (3441)	12,132 (4337)^a^	13,837 (4457)^b,c^*	*0.94*	9048 (2683)	9851 (2948)	11,132 (3251)^b,c^*	*0.70*
Self-reported screen time (h/day)	3.6 (2.3)	2.8 (1.8)^a^	2.6 (1.9)^c^*	*0.47*	3.0 (1.9)	2.5 (1.9)	2.1 (1.5)^c^*	*0.52*
Average days/week meeting the guidelines	4.4 (2.1)	4.9 (1.8)	5.6 (1.7)^b,c^*	*0.63*	4.4 (1.7)	4.8 (1.9)	5.4 (1.5)^b,c^*	*0.62*
Motivation and Confidence domain								
Total domain score	11.0 (3.5)	12.7 (2.8)^a^	14.4 (2.1)^b,c^*	*1.18*	11.2 (2.9)	12.2 (2.6)^a^	13.5 (2.3)^b,c^*	*0.88*
Benefits and barriers	1.3 (1.4)	1.5 (1.2)	2.1 (1.0)^b,c^*	*0.66*	1.2 (1.2)	1.5 (1.1)	1.8 (1.0)^c^*	*0.54*
Activity levels compared to peers	0.6 (0.2)	0.7 (0.2)^a^	0.8 (0.1)^b,c^*	*1.27*	0.6 (0.2)	0.7 (0.2)	0.8 (0.2)^b,c^*	*1.00*
Skill level compared to peers	0.6 (0.3)	0.7 (0.2)^a^	0.8 (0.2)^b,c^*	*0.79*	0.6 (0.2)	0.6 (0.2)^a^	0.7 (0.2)^b,c^*	*0.50*
CSAPPA adequacy score	4.3 (1.1)	4.9 (0.9)^a^	5.3 (0.6)^b,c^*	*1.13*	4.3 (0.9)	4.6 (0.9)^a^	5.1 (0.8)^b,c^*	*0.94*
CSAPPA predilection score	4.2 (1.2)	4.9 (1.0)^a^	5.3 (0.7)^b,c^*	*1.12*	4.4 (1.1)	4.8 (1.0)^a^	5.1 (0.8)^b,c^*	*0.73*
Knowledge and Understanding domain								
Total domain score	11.6 (2.8)	13.1 (2.4)^a^	13.4 (2.3)^c^*	*0.70*	12.4 (2.7)	13.2 (2.4)^a^	13.6 (2.2)^c^*	*0.49*
CAPL questionnaire score	11.6 (2.8)	13.1 (2.4)^a^	13.4 (2.3)^c^*	*0.70*	12.4 (2.7)	13.2 (2.4)^a^	13.6 (2.2)^c^*	*0.49*

Note: All variables are presented as means (±SD). Statistical significance for the main effect was assessed using ANCOVA controlling for maturity offset. Bonferroni (Dunn) t-test was used to assess differences between groups. Cohen’s *d* was used to calculate the effect size between the low and high CRF groups

*20mSRT* 20-m shuttle run test, *ANCOVA* analysis of covariance, *BMI* body mass index, *CAMSA* Canadian Agility and Movement Skill Assessment, *CAPL* Canadian Assessment of Physical Literacy, *CRF* cardiorespiratory fitness, *CSAPPA* Children’s Self-Perception and Adequacy in and Predilection for Physical Activity, *SD* standard deviation

^a^*p* < 0.05 for medium CRF vs low CRF

^b^*p* < 0.05 for high CRF vs medium CRF

^c^*p* < 0.05 for high CRF vs low CRF

*p < 0.001 for main effect

## Discussion

This study represents the largest effort to date to assess the associations between CRF and components of PL among school-aged children. Our findings suggest that there are clear favourable associations between PL and CRF levels. For instance, participants in the high CRF tertile consistently demonstrated better scores across all domains of PL in comparison with their peers in lower CRF tertile groups, regardless of age and gender. Of the PL components, the strongest associations were identified between CRF and Physical Competence (large effect size), followed by Motivation and Confidence (moderate to large effect size), Daily Behaviour (small to moderate effect size), and Knowledge and Understanding (marginal to moderate effect size).

### Associations across components of physical literacy

Our study identifies consistent and favourable gradients across CRF tertiles for all components that describe PL. This finding suggests that CRF, measured using the 15mSRT or 20mSRT, is an important correlate of all PL domains and almost all components of the CAPL among Canadian children aged 8–12 years, supporting a growing body of literature that highlights the importance of CRF in these age groups [32].

This study identifies strong associations between CRF and most components of the Physical Competence domain, which supports the literature [10]. Of the components that describe Physical Competence, the largest effect sizes across age and gender groups were identified for the CAMSA (i.e., gross motor skills) and the prone plank test (i.e., muscular endurance). The CAMSA and the prone plank test are novel assessments used in the CAPL and this study is the first to identify these strong associations; however, other studies using different tests support our findings. For instance, a previous study identified strong positive associations between the Test of Gross Motor Development–2nd Edition and achieving the FITNESSGRAM® Healthy Fitness Zone for the 20mSRT [33, 34]. This further suggests that CRF is strongly related to gross motor skills. In addition, another study reported marginal but significant correlations between CRF and the ability to perform repeated push-ups and curl-ups [35], measures of muscular endurance that are similar to the prone plank test. These associations suggest that future interventions designed to improve CRF could incorporate skill development aimed at teaching fundamental motor skills (i.e., jumping, sliding, catching, throwing, skipping, hopping, and kicking), movement capacities (i.e., coordination, balance, precision, acceleration, and deceleration), and muscular endurance. These types of interventions may have an indirect or mediated effect by providing children with the abilities needed to participate in a broader range of physical activities, and thus potentially further improve CRF. Indeed, many physical education programs incorporate these aspects of skill development in their respective curricula.

In our study, the association between CRF and adiposity (body mass index and waist circumference) ranged from moderate to large. While the relationship between CRF and adiposity is certainly important, it may not be the most vital aspect associated with 20mSRT performance. Previous research has showed that adiposity explained between 40 and 60% of declines in distance running seen in children (aged 10–12 years) between 1985 and 1997 [36]. Indeed, 20mSRT performance in children likely results from a combination of several physiological and psychological aspects, including motivation [37].

The importance of motivation is further supported by our study, which identified the Motivation and Confidence domain as having moderate to large effect sizes across age and gender groups. This is noteworthy given that the range of effect sizes for the Motivation and Confidence domain was stronger than the overall Daily Behaviour domain across most age and gender groups. Thus, our findings suggest that creating a motivational climate (e.g., mastery climate, achievement goal theory, self-determination theory, etc.) that encourages increased effort and task motivation could positively impact CRF, and the context in which children are active (e.g., encouragement, reinforcement, etc.) may be more important than simply getting children moving.

### Strategies for CRF and PL surveillance

Our study identified strong associations between CRF and PL that generally increased with age. Given this finding, the 20mSRT could have merit as a simple screening assessment to help identify children with low PL and in need of a full CAPL assessment, providing a way to save time and resources in the school-based setting. This strategy is consistent with other studies that have advocated for the use of the 20mSRT as a population health surveillance instrument to help identify children and youth at risk of poor health outcomes [9]. This study also highlights the possibility of developing new PL-based CRF criterion-referenced standards for children that would incorporate a variety of cognitive, mental, and physical health indicators that may help screen children at increased risk of lifestyle-related disease.

Although this study has identified the potential of the 20mSRT as a screening instrument for PL in the school-based environment, there is still more work to be done. For instance, future studies are needed to better understand the sensitivity and specificity of using CRF cut-off values to identify those with low PL levels.

### Strengths and limitations

An important strength of this study is the large sample of Canadian children tested using a standardized data collection protocol, validated CAPL instruments, and trained assessors across all 11 sites. We collected data during a relatively small time frame (i.e., 3 years), thereby limiting the possible effect of temporal trends. We also aimed to obtain a balanced sample across Eastern and Western Canada to diminish any potential within-country differences in our estimates. However, our results need to be interpreted in light of the following limitations. First, this was a cross-sectional study and therefore causality cannot be inferred. Second, in light of the voluntary and purposive sampling strategy, the results may not be generalizable to the broader Canadian context. However, portions of the Physical Competence (i.e., grip strength, waist circumference, body mass index) results from our 8- to 12-year-old sample were only slightly higher to a published national representative sample of Canadian children aged 6–10 years [38]. Third, significant heterogeneity of variance was observed in 50% of the ANCOVA analyses, which violates the assumption of equality of variance. However, ANCOVA is generally robust to moderate violations of heterogeneity of variance, as long as the sample size is large and approximately equal across groups [39], which was true for our study. Finally, the potential confounding effects of unmeasured variables (e.g., measured maturation, socio-economic status, ethnicity, etc.) cannot be discounted. Although we have made an attempt to predict maturity offset using the Moore et al. equation [30], these predicted estimates may not have been accurate as they were predicted using age and standing height as inputs.

## Conclusions

This study identified strong favourable associations between CRF and all components of PL in a large sample of school-aged Canadian children. These findings provide preliminary evidence to support the importance of CRF as a possible predictor of PL outcomes. Future studies should aim to replicate these results in different populations, and to identify the sensitivity and specificity of using CRF to screen for children with low PL levels.

## Additional file

Additional file 1: Percentages of Canadian boys and girls with healthy cardiorespiratory fitness using the interim international standards [16]. Data are presented as % and standard deviations. (BMP 1574 kb)

## Abbreviations

20mSRT: 20 m shuttle run test
ANCOVA: Analysis of covariance
CAMSA: Canadian Agility and Movement Skill Assessment
CRF: Cardiorespiratory fitness
CSAPPA: Children’s Self-Perception and Adequacy in and Predilection for Physical Activity
PL: Physical literacy
R^2^: R-squared
RBC-CAPL: Royal Bank of Canada–Canadian Assessment of Physical Literacy Learn to Play project
SD: Standard deviation
SEE: Standard error of the estimate

## References

[1] Lloyd M, Colley RC, Tremblay MS (2010). Advancing the debate on ‘fitness testing’ for children: perhaps we’re riding the wrong animal. Pediatr Exerc Sci.

[2] Whitehead M (2013). Definition of physical literacy and clarification of related issues. ICSSPE Bull.

[3] Longmuir PE, Boyer C, Lloyd M, Yang Y, Boiarskaia E, Zhu W (2015). The Canadian assessment of physical literacy: methods for children in grades 4 to 6 (8 to 12 years). BMC Public Health.

[4] Francis CE, Longmuir PE, Boyer C, Andersen LB, Barnes JD, Boiarskaia E (2016). The Canadian assessment of physical literacy: development of a model of children’s capacity for a healthy, active lifestyle through a Delphi process. J Phys Act Health.

[5] Léger L, Lambert J, Goulet A, Rowan C, Dinelle Y (1984). Capacité aérobie des Québécois de 6 à 17 ans – test navette do 20 mètre avec paliers do 1 minute. Can J Appl Sport Sci.

[6] Léger LA, Mercier D, Gadoury C, Lambert J (1988). The multistage 20 metre shuttle run test for aerobic fitness. J Sports Sci.

[7] Mayorga-Vega M, Aguilar-Soto P, Viciana J (2015). Criterion-related validity of the 20-m shuttle run test for estimating cardiorespiratory fitness: a meta-analysis. J Sports Sci Med.

[8] Ortega F B, Artero E G, Ruiz J R, Vicente-Rodriguez G, Bergman P, Hagströmer M, Ottevaere C, Nagy E, Konsta O, Rey-López J P, Polito A, Dietrich S, Plada M, Béghin L, Manios Y, Sjöström M, Castillo M J (2008). Reliability of health-related physical fitness tests in European adolescents. The HELENA Study. International Journal of Obesity.

[9] Lang JJ, Tremblay MS, Léger L, Olds TS, Tomkinson GR. International variability in 20 m shuttle run performance in children and youth: who are the fittest from a 50-country comparison? A systematic literature review with pooling of aggregate results. Br J Sports Med. Published online first: 20 Sept 2016. 10.1136/bjsports-2016-096224 27650256

[10] Dumith SC, van Dusen D, Kohl HW (2012). Physical fitness measures among children and adolescents: are they all necessary?. J Sports Med Phys Fitness.

[11] Kristensen PL, Moeller NC, Korsholm L, Kolle E, Wedderkopp N, Froberg K (2010). The association between aerobic fitness and physical activity in children and adolescents: the European youth heart study. Eur J Appl Physiol.

[12] Williams SM, Phelps D, Laurson KR, Thomas DQ, Brown DD (2013). Fitness knowledge, cardiorespiratory endurance and body composition of high school students. Biomed Hum Kinet.

[13] Sardinha LB, Marques A, Martins S, Palmeira A, Minderico C (2014). Fitness, fatness, and academic performance in seventh-grade elementary school students. BMC Pediatr.

[14] Colquitt G, Walker A, Langdon JL, McCollum S, Pomazal M (2012). Exploring student attitudes toward physical education and implications for policy. Sport Sci Pract Aspects.

[15] Goa Z, Lodewyk KR, Zhang T (2009). The role of ability beliefs and incentives in middle school students’ intention, cardiovascular fitness, and effort. J Teach Phys Educ.

[16] Ruiz JR, Cavero-Redondo I, Ortega FB, Welk GJ, Andersen LB, Martinez-Vizcaino V. Cardiorespiratory fitness cut points to avoid cardiovascular disease risk in children and adolescents; what level of fitness should raise a red flag? A systematic review and meta-analysis. Br J Sports Med. Published online first: 26 September 2016. 10.1136/bjsports-2015-095903 27670254

[17] Lang JJ, Tremblay MS, Ortega FB, Ruiz JR, Tomkinson GR. Review of criterion-referenced standards for cardiorespiratory fitness: what percentage of 1 142 026 international children and youth are apparently healthy? Br J Sports Med. Published online first: 2 Jan 2017. 10.1136/bjsports-2016-096955 28254744

[18] McClain JJ, Welk GJ, Ihmels M, Schaben J (2006). Comparison of two versions of the PACER aerobic fitness test. J Phys Act Health.

[19] Tomkinson GR, Lang JJ, Tremblay MS, Dale M, LeBlanc AG, Belanger K, et al. International normative 20 m shuttle run values from 1 142 026 children and youth representing 50 countries. Br J Sports Med. Published online first: 23 April 2016. 10.1136/bjsports-2016-095987 27208067

[20] Tremblay MS, Shields M, Laviolette M, Craig CL, Janssen I, Gorber SC (2010). Fitness of Canadian children and youth: results from the 2007–2009 Canadian health measures survey. Health Rep.

[21] Boyer C, Tremblay MS, Saunders TJ, McFarlane A, Borghese M, Lloyd M, Longmuir P (2013). Feasibility, validity, and reliability of the plank isometric holds as a field-based assessment of torso muscular endurance for children 8 to 12 years of age. Pediatr Exerc Sci.

[22] Longmuir PE, Boyer C, Lloyd M, Andersen LB, Barnes JD, Boiarskaia E, et al. Canadian Agility and Movement Skill Assessment (CAMSA): validity, objectivity, and reliability evidence for children 8–12 years of age. J Sport Health Sci. Published online first: 11 November 2015. 10.1016/j.jshs.2015.11.004 PMC6189007 30356598

[23] Saunders TJ, Gray CE, Borghese MM, McFarlane A, Mbonu A, Ferraro ZM (2014). Validity of SC-StepRx pedometer-derived moderate and vigorous physical activity during treadmill walking and running in a heterogeneous sample of children and youth. BMC Public Health.

[24] Tudor-Locke C, McClain JJ, Hart TL, Sisson SB, Washington TL (2009). Expected values for pedometer-determined physical activity in youth. Res Q Exerc Sport.

[25] Brener ND, Kann L, Shanklin S, Kinchen S, Eaton DK, Hawkins J, Centers for Disease Control and Prevention (2013). Methodology of the Youth Risk Behavior Surveillance System – 2013. MMWR Recomm Rep..

[26] Garcia AW, Broda MA, Frenn M, Coviak C, Pender NJ, Ronis DL (1995). Gender and developmental differences in exercise beliefs among youth and prediction of their exercise behavior. J Sch Health.

[27] Hay JA (1992). Adequacy in and predilection for physical activity in children. Clin J Sports Med.

[28] Tremblay MS, Carson V, Chaput JP, Connor Gorber S, Dinh T, Duggan M (2016). Canadian 24-hour movement guidelines for children and youth: an integration of physical activity, sedentary behaviour, and sleep. Appl Physiol Nutr Metab.

[29] Physical Activity Guidelines: children and adolescents. Office of Disease Prevention and Health Promotion. http://health.gov/paguidelines/guidelines/children.aspx. Accessed 17 July 2017.

[30] Moore SA, Mckay HA, Macdonald H, Nettlefold L, Baxter-Jones ADG, Cameron N (2015). Enhancing a somatic maturity prediction model. Med Sci Sports Exerc.

[31] Cohen J (1988). Statistical power analysis for the behavioral sciences.

[32] Ortega FB, Ruiz JR, Castillo MJ, Sjöström M (2008). Physical fitness in childhood and adolescence: a powerful marker of health. Int J Obes.

[33] Burns RD, Brusseau TA, Fu Y, Hannon JC (2015). Predictors and trends of gross motor skill performance in at-risk elementary school-aged children. Percept Mot Skills.

[34] Welk GJ, Laurson KR, Eisenmann JC, Cureton K (2011). Development of youth aerobic-capacity standards using receiver operating characteristic curves. Am J Prev Med.

[35] Coe DP, Peterson T, Blair C, Schutten MC, Peddie H (2013). Physical fitness, academic achievement, and socioeconomic status in school-aged youth. J School Health.

[36] Olds TS, Dollman J (2004). Are changes in distance-run performance of Australian children between 1985 and 1997 explained by changes in fatness?. Pediat Exerc Sci.

[37] Tomkinson GR, Olds TS, Armstrong N, van Mechelen W (2000). Field tests of fitness. Paediatric exercise science and medicine.

[38] Tremblay MS, Shields M, Laviolette M, Craig CL, Janssen I, Connor GS (2010). Fitness of Canadian children and youth: results from the 2007–2009 Canadian health measures survey. Health Rep.

[39] Nimon KF (2012). Statistical assumptions of substantive analyses across the general linear model: a mini-review. Front Psychol.

