# Supplementary figures and images for: Cardiorespiratory fitness is associated with physical literacy in a large sample of Canadian children aged 8 to 12 years
Source: BMC Public Health. 2018 Oct 2;18(Suppl 2):1041. doi: 10.1186/s12889-018-5896-5 (PMC6167777; doi:10.1186/s12889-018-5896-5)

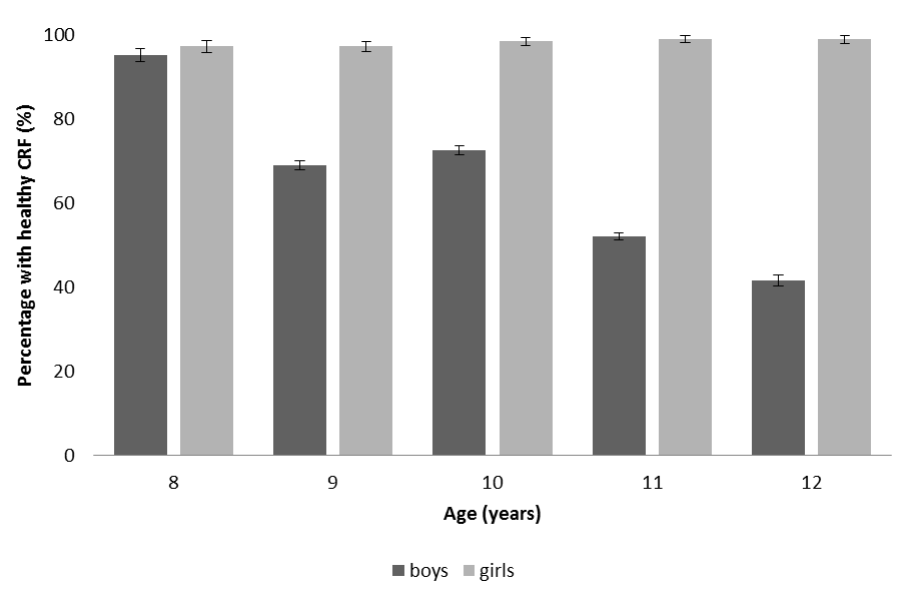

Supplement: Supplementary file 1 — Percentages of Canadian boys and girls with healthy cardiorespiratory fitness using the interim international standards [16]. Data are presented as % and standard deviations. (BMP 1574 kb) [file 12889_2018_5896_MOESM1_ESM.bmp]
